# Use of angiotensin II receptor blocker during pregnancy: A case report

**DOI:** 10.1097/MD.0000000000024304

**Published:** 2021-01-22

**Authors:** Qiang Wei, Li Zhang, Mei-fan Duan, Yue-mei Wang, Nan Huang, Chun-rong Song

**Affiliations:** Department of Obstetrics and Gynecology, Key Laboratory of Birth Defects and Related Diseases of Women and Children of the Ministry of Education, West China Second University Hospital, Sichuan University, Chengdu, People's Republic of China.

**Keywords:** angiotensin II receptor blockers, hypertension, losartan, oligohydramnios, perinatal outcome

## Abstract

**Background:**

Drugs that affect the renin-angiotensin system, such as angiotensin II receptor blockers (ARBs) and angiotensin-converting enzyme inhibitors are not typically recommended for pregnant women because of their potential fetal toxicity.

**Case study:**

A 32-year-old pregnant woman with nephrotic syndrome lasting more than 5 years became pregnant for the first time. She had been taking losartan tablets before and during pregnancy. Ultrasound at 24^+2^ weeks of pregnancy showed oligohydramnios, and the maximum vertical depth of amniotic fluid volume was 1.4 cm. Follow-up ultrasound examinations every 2 weeks showed persistent oligohydramnios [amniotic fluid volume: 1.1–3.4 cm, amniotic fluid index 1.9–6.9 cm]. B-ultrasound at 30^+2^ weeks showed slightly enhanced fetal renal cortex echo. The patient was treated at 32^+2^ weeks of pregnancy at our hospital.

**Diagnoses:**

Nephrotic syndrome and oligohydramnios.

**Interventions:**

Losartan was discontinued and replaced by nifedipine controlled-release tablets to lower blood pressure. The amount of amniotic fluid gradually increased to normal levels within 8 days. The patient was discharged at 33^+2^ weeks of pregnancy for follow-up. At 34^+4^ weeks, blood pressure had increased to 177/113 mm Hg and the patient was re-hospitalized with nephrotic syndrome complicated by preeclampsia. Due to progression of severe preeclampsia, elective cesarean section was performed at 35^+3^ weeks. After delivery, losartan and nifedipine were prescribed to continue lowering blood pressure. The patient was discharged 4 days after surgery.

**Outcomes:**

Losartan use was terminated at 32^+2^ weeks of pregnancy. Amniotic fluid returned to normal after 8 days and the baby was delivered after 22 days. At last follow-up, the infant was 24 months old and healthy.

**Conclusion:**

Although ARBs are effective for treating hypertension, they should be replaced by other classes of anti-hypertensive drugs in pregnant women. Pregnant women who elect to continue using ARBs should be informed about risks, they should be carefully monitored during pregnancy, and their pregnancy should be allowed to proceed as long as clinically feasible in order to optimize maternal and infant outcomes.

## 1 Introduction

The renin-angiotensin system plays an important role in regulating blood pressure and homeostasis. Drugs that interfere with this system, such as angiotensin-converting enzyme inhibitors (ACEIs), and angiotensin receptor blockers (ARBs) are widely used in the treatment of hypertension and other cardiovascular diseases.^[1]^ In particular, ARB use may be increasing worldwide.^[1]^ Previous studies and pharmacopoeiae recommend that ACEIs and ARBs not be used during pregnancy because of their potential fetal toxicity.^[1,2]^ Nevertheless, some patients continue to use such drugs during pregnancy, and patients can wonder whether they should terminate their pregnancy because of their previous use of such drugs. Recently, a pregnant woman who took ARBs before and through a late trimester of pregnancy was admitted to our hospital. We describe this case and review relevant literature in order to highlight the problems associated with the use of ARB drugs during pregnancy.

## 2 Case study

A 32-year-old Chinese female patient with nephrotic syndrome lasting more than 5 years got pregnant for the first time (G1P0) duringtreatments with losartan potassium tablets (50 mg q.d.), methylprednisolone tablets (12 mg q.o.d.), and dipyridamole tablets (50 mg q.d.). She continued these drgs and had regular outpatient check-ups during pregnancy. Non-invasive DNA prenatal screening, 75-g oral glucose tolerance test, and fetal cardiac ultrasound were performed and were normal. Fetal systemic ultrasound at 24^+2^ weeks of pregnancy indicated oligohydramnios for the first time (The sonographic diagnosis of oligohydramnios is based on the amniotic fluid index ≤ 5 cm or the maximum vertical depth of amniotic fluid volume (AFV)≤ 2 cm.^[3]^), the AFV was 1.4 cm. Follow-up ultrasound examinations every 2 weeks showed that AFV ranged 1.1 to 3.4 cm, and amniotic fluid index was 1.9 to 6.9 cm. B-scan ultrasonography at 30^+2^ weeks of pregnancy indicated that fetal renal cortex echo was slightly enhanced. At outpatient checkups during pregnancy, systolic blood pressure fluctuated from 103 mm Hg to 153 mm Hg and diastolic pressure from 89 to 118 mm Hg; self-monitoring at home indicated relatively stable, normal blood pressure around 130/80 mm Hg. Routine urinalysis during pregnancy was normal, and renal function and serum albumin were normal.

The patient was transferred to our hospital for oligohydramnios at 32^+2^ weeks of pregnancy. Blood pressure was 130/91 mm Hg. The uterus was 29 cm high, abdominal circumference was 101 cm, and fetal head position and heart were normal. No contractions were detected. Immediately after admission, based on the recommendation from our nephrology department, losartan and methylprednisolone were discontinued and replaced by oral nifedipinecontrolled-release tablets (30 mg q.d.) to lower blood pressure. Blood pressure and amniotic fluid were monitored for 8 days. Systolic blood pressure fluctuated from 126 to 150 mm Hg; diastolic, from 72 to 97 mm Hg. The amount of amniotic fluid gradually increased to normal levels. The patient was discharged at 33^+2^ weeks of pregnancy for follow-up. Ultrasound findings of the fetus and amount of amniotic fluid during pregnancy are shown in Table 1. At 34^+4^ weeks of pregnancy, her blood pressure rosed to 177/113 mm Hg at an outpatient check-up. The patient was re-admitted to the hospital and diagnosed with nephrotic syndrome complicated by preeclampsia, and G1P0 intrauterine pregnancy at 34^+4^ weeks.

**Table 1:** Ultrasound findings of the fetus during pregnancy.

Gestational age (wk)	BPD (cm)	FL (cm)	HC (cm)	AC (cm)	AFV (cm)	AFI (cm)	UA-S/D	Remarks
24^+2^	6.35	4.28	22.40	20.22	1.4	-	3.54	Left choroid plexus cyst 0.4 × 0.3 cm
26^+2^	6.49	4.78	23.72	22.72	3.4	6.8	3.61	Left choroid plexus cyst 0.3 × 0.2 cm
28^+2^	7.33	5.20	26.41	23.18	3.1	6.9	3.42	Normal
30^+2^	7.48	5.60	27.6	25.95	2.3	4.5	2.50	Large kidneys with slightly enhanced parenchymal echo
32^+2^	8.09	6.27	29.47	27.04	1.1	1.9	2.80	Echoes of both renal cortices slightly enhanced
32^+6^	8.02	6.16	28.90	27.90	2.6	4.3	1.87	Echoes of both renal cortices slightly enhanced
33^+2^	8.18	6.42	29.12	28.70	3.0	6.4	2.86	Echoes of both renal cortices slightly enhanced
33^+5^	8.21	6.43	29.40	28.90	3.0	7.4	2.18	Echoes of both renal cortices slightly enhanced
34	8.31	6.50	30.05	29.25	3.6	7.9	2.80	Echoes of both renal cortices slightly enhanced
35^+2^	8.30	6.80	30.00	32.90	4.8	9.1	2.10	Echoes of both renal cortices slightly enhanced

After this re-admission, the patient was treated with magnesium sulfate (IV infusion at a speed of 1 g/h) with added nifedipinecontrolled-release tablets (60 mg q.d.) and additionallabetaloltablets (200 mg b.i.d.) to lower blood pressure. During the following 4 days, proteinuria increased from (+) to (++), 24-h urinary protein levels increased from 3.51 g to 9.19 g, and urinary casts became visible. Due to nephrotic syndrome, and progression of severe preeclampsia, elective cesarean section was performed at 35^+3^ weeks of pregnancy. During the operation, about 500 mL of clear amniotic fluid was observed, and the newborn was a male, 43 cm long and weighing 2200 g. Neonatal Apgar score were both 10 at 1 and 5 minute, and no birth defects were found After consultation with the nephrology department, the patient was prescribed losartan (100 mg q.d.), nifedipine (30 mg b.i.d.), and methylprednisolone (28 mg q.d.). The patient showed stable vital signs after surgery, and systolic blood pressure fluctuated between 130 and 145 mm Hg; diastolic, between 75 and 92 mm Hg. The patient was discharged 4 days after delivery.

Ultrasound examination and renal testing of the newborn immediately after birth revealed no abnormality. At 3 days after birth, the infant was transferred to the neonatal ward because of neonatal jaundice and discharged 5 days afterwards. At the last follow-up, the 24-month-old baby was 86 cm tall, weighed 12.5 kg, and showed normal intellectual and physical development.

## 3 Discussion

### 3.1 Selection of antihypertensive drugs during pregnancy

Renin-angiotensin inhibitors such as ACEIs and ARBs are the antihypertensive drugs of choice for hypertensive patients who are not pregnant,^[4–6]^ including nearly 50% of women of childbearing age.^[7,8]^ However, given the potential fetal toxicity of these drugs, their use during pregnancy is not recommended.^[1,2]^ Instead, the New Zealand Ministry of Health (2018) and the American College of Obstetricians and Gynecologists (2019) recommend calcium channel blockers (such as nifedipine), beta receptor blockers (such as labetalol), methyldopa, and hydralazine for pregnancy hypertension.^[2,9]^ For patients with chronic hypertension complicated with pregnancy, the International Society for the Study of Hypertension in Pregnancy in their 2018 guidelines recommends first-line drugs including labetalol, propranolol, methyldopa, nifedipine, and diltiazem, while prazosin and hydralazine should be used as second- or third-line antihypertensives.^[10]^

### 3.2 ARBs mechanism of action

Angiotensin (Ang) II is the main effector hormone of the renin-angiotensin-aldosterone system and has multiple functions. ARBs selectively block 1 of the 2 Ang II receptors, namely AT1R, thereby blocking the abnormally activated renin-angiotensin-aldosterone system. ARBs inhibit vasoconstriction, reduce peripheral vascular resistance, and inhibit aldosterone secretion, reducing water and sodium retention.^[1]^ ARBs act at the receptor level to block the renin-angiotensin-aldosterone system more directly than ACEIs, allowing them to avoid “Ang II escape”.^[1]^ ARBs show better antihypertensive efficacy and fewer side effects than ACEIs and have become first-line antihypertensive drugs widely used in clinical practice.^[2]^

### 3.3 Adverse effects of ARBs during pregnancy

The renin-angiotensin-aldosterone system plays a vital role in regulating blood pressure, water, and salt balance; it also controls renal hemodynamics and glomerular filtration.^[11]^ This system is very important for the development of the kidneys in newborns. Fetal kidneys begin to develop in the 5^th^ week of pregnancy, and urine begins to be produced around the 9^th^ to 12^th^ week.^[12]^ Nephrons are actively formed throughout fetal growth, starting from about 15,000 nephrons per kidney at 15 weeks of gestation, to 1 million per kidney at 40 weeks.^[13]^ The glomerular filtration rate gradually increases from 34 to 36 weeks of gestation, when kidney development is complete. Glomerular filtration continues to mature early in the postnatal period, and the rate doubles during the first few weeks.^[14]^ If the kidneys do not develop fully during the fetal period, renal perfusion and glomerular filtration may be quite sensitive to exogenous factors.

Studies have reported adverse fetal outcomes after maternal use of ARBs, including renal failure, lung dysplasia, cranial hypoplasia, limb contractures, and fetal or neonatal death. These outcomes may be due to fetal hypotension and reduced renal blood flow perfusion, resulting in kidney ischemia, anuria, and oligohydramnios.^[15]^ Oligohydramnios may cause limb contracture, craniofacial deformity, and lung dysplasia. ARBs may also reduce placental and umbilical cord blood perfusion, leading to fetal growth restriction and skull angiogenesis that affects skull ossification.^[16]^

The safety of renin-angiotensin-aldosterone inhibitors in early pregnancy continues to be debated, with only limited clinical data available. Epidemiological studies have associated ACEI or ARB use by pregnant women with increased incidence of congenital anomalies.^[17]^ However, a similar increase in congenital anomalies has been reported among women taking other anti-hypertensives or no such drugs at all.^[17]^ This finding suggests that the observed teratogenicity is likely to be linked to maternal factors and comorbidities coexisting with hypertension during pregnancy, such as diabetes, older age, or obesity.^[17]^ A cohort study comparing outcomes of 215 pregnancies involving ARB exposure during the first trimester to 642 non-hypertensive pregnancies showed that the rate of major birth defects was marginally but not significantly higher in the ARB cohort [5.4% (9/168) vs 3% (17/570)] ^[18]^, and ARB use was not associated with higher risk of spontaneous abortions or preterm birth. There was no distinct pattern of anomalies among infants with birth defects. Risk of birth defects was higher for pregnancies in which ARB exposure continued after gestational week 6 than for pregnancies in which ARBs were discontinued before gestational week 6 [7.3% (7/96) vs 2.8% (2/72)]. This may point to a teratogenic vulnerability in the second half of the first trimester. While this study supports the hypothesis that ARBs are not major teratogens, women planning pregnancy should nevertheless avoid ARBs.

Our patient was initially admitted to our hospital because of oligohydramnios. Whether oligohydramnios can be corrected in pregnant women who resume taking ARBs after discontinuing them is an open question, as is whether correcting for oligohydramnios improves fetal pregnancy outcomes. A systematic review found high incidence of oligohydramnios with ARB use in early pregnancy (43%) and even higher incidence (87%) when the drug was used in mid-to-late pregnancy or throughout pregnancy.^[19]^ Review of 63 pregnancies in which the mothers used ARBs in the second and third trimesters ^[20]^ found that discontinuing ARBs improved oligohydramnios; in fact, fetal outcomes were better when ARBs were discontinued earlier and the interval between drug withdrawal and delivery was longer, especially more than 3 weeks. In our case, the patient used losartan until the third trimester of pregnancy and discontinued at 32^+2^ weeks of pregnancy. The amount of amniotic fluid returned to normal after 8 days of discontinuation, and the pregnancy was terminated 22 days after discontinuation at 35^+3^ weeks. At the last follow-up, the 24-month-old baby was 86 cm tall, weighed 12.5 kg, and showed normal intellectual and physical development. Based on the literature, the good maternal and fetal outcomes in our case may be attributed to the fact that the amount of amniotic fluid in our patient returned to normal after ARB discontinuation, and the fact that delivery occurred more than 3 weeks after ARB discontinuation.

The present case supports the literature and official guidelines that highlight the risks associated with ARB use by pregnant women. Pregnant women should be advised to change to other anti-hypertensive medications as soon as possible. If they elect to continue taking ARBs, they should be fully informed about the relevant risks based on current research and comprehensive consideration of the patient's condition. If the patient chooses to continue pregnancy, the pregnancy should be monitored carefully and allowed to proceed for as long as clinically feasible, in order to optimize maternal and infant outcomes.

## Author contributions

Qiang Wei drafted the manuscript, collected and analyzed the data. Li Zhang conceived and designed the study, helped draft the manuscript, revised it critically for important intellectual content, and coordinated data collection. All authors read and approved the final manuscript.

**Conceptualization:** Qiang Wei, li zhang.

**Data curation:** Qiang Wei, Mei-fan Duan, Yue-mei Wang, Nan Huang.

**Formal analysis:** Li Zhang.

**Methodology:** Li Zhang.

**Supervision:** Li Zhang.

**Writing – original draft:** Qiang Wei, li zhang, Chun-rong Song.

**Writing – review & editing:** Li Zhang.
